# Precise balloon pressure regulation in percutaneous balloon compression for trigeminal neuralgia: evidence from a single-center prospective cohort

**DOI:** 10.3389/fsurg.2026.1651583

**Published:** 2026-03-10

**Authors:** Fei Wang, Kun Guo, Qihao Sun, Jian Zhang, Bingwen Yu, ShaoPeng Peng

**Affiliations:** 1 Department of Neurointervention, Shaoguan First People's Hospital, Affiliated Hospital of Shaoguan University, Shaoguan, Guangdong, China; 2 Department of Neurosurgery, Gansu Provincial People’s Hospital, Lanzhou, Gansu, China; 3 Department of Neurology, Xi'an Central Hospital, Xi'an, Shaanxi, China

**Keywords:** balloon pressure, percutaneous balloon compression, postoperative complications, precision surgery, trigeminal neuralgia

## Abstract

**Background:**

Trigeminal neuralgia (TN) causes severe paroxysmal facial pain and quality-of-life impairment. Percutaneous balloon compression (PBC) is minimally invasive, yet efficacy and complication rates remain variable. We conducted a single-centre prospective cohort study to define phase-specific intraballoon pressure thresholds that maximize pain relief while minimising facial hypoaesthesia and recurrence.

**Methods:**

115-TN patients undergoing PBC (Jan 2017–Aug 2024) had real-time pressure recorded at 1 Hz. Pressure morphometrics were classified into pear-shaping, maintenance and full-compression phases. Reference intervals (RI) were established with the non-parametric CLSI C28-A2 method (*n* ≥ 4,000 measurements per phase). Associations between phase pressures and 24-h hypoaesthesia grade or 24-month recurrence were examined with Spearman correlation and Cox regression (Schoenfeld validation).

**Results:**

Higher phase pressures correlated with more severe hypoaesthesia (*ρ* = 0.44–0.62, *P* < 0.01), whereas lower pressures predicted increased recurrence (HR = 0.87 per 5 kPa, 95% CI 0.80–0.94, *P* = 0.002). Optimal RI were: pear-shaping 126.5–156.8 kPa, maintenance 117.9–136.1 kPa, full-compression 119.9–141.9 kPa. Operating within these bands produced BNI-I/II pain relief in 98% of patients with only 16% transient Grade-III hypoaesthesia.

**Conclusion:**

Real-time, phase-adapted pressure control within the proposed RI allows surgeons to standardize PBC while individualizing the therapeutic window, improving safety and durability of pain relief.

## 1 Introduction

Trigeminal Neuralgia (TN) is a neuropathic pain disorder marked by severe facial pain, which can considerably reduce the patient's quality of life (1). Percutaneous balloon compression (PBC), initially described by Mullan and Lichtor in 1983, achieves mechanical ablation of the trigeminal ganglion through controlled balloon inflation (2). Compared with microvascular decompression (MVD), PBC offers distinct advantages including shorter operative duration, reduced morbidity, and minimal invasiveness (3, 4). These attributes render it particularly suitable for elderly patients, those medically unfit for prolonged general anaesthesia, or individuals declining open cranial surgery (5, 6). Through iterative refinements, PBC has evolved into a widely adopted minimally invasive intervention for TN.

PBC outcomes are influenced by multiple technical factors, including balloon morphology, volume, compression duration, and pressure (7, 8). Current consensus indicates optimal efficacy when the balloon assumes a “pear-shaped” morphology intraoperatively, with a compression duration of 120–180 s yielding favorable results. Sun et al (9). evaluated over 150 PBC cases with two-year follow-up, reporting near-complete pain resolution (approaching 100%) when lateral fluoroscopy confirmed pear-shaped balloon formation. This finding aligns with conclusions by Pär Asplund et al. Notably, Huo et al. utilised Dyna-CT reconstruction to demonstrate superior outcomes with balloon volumes of 568.2–891.4 mm^3^, correlating with an elliptical morphology (10). Regarding compression duration, most studies support 1–3 min as clinically effective. Some surgeons advocate 60 s as a minimum threshold for adequate analgesia (11, 12). This is corroborated by animal studies showing effective nociceptive blockade in Oryctolagus cuniculus (rabbit) models at 2-minute compression (13).

While optimal pear-shaped morphology, appropriate balloon volume, and precise compression duration constitute necessary prerequisites for percutaneous balloon compression (PBC), intraoperative pressure regulation emerges as the paramount factor governing therapeutic efficacy and safety (14). Excessive pressures (>250 kPa) precipitate balloon rupture with contrast extravasation, significantly elevating risks of postoperative hypoaesthesia and masticatory weakness. Conversely, inadequate pressures (<130 kPa) compromise pain relief and heighten recurrence rates. Empirical evidence substantiates this paradigm: Brown et al. documented a mean intraballoon pressure of 109.5 ± 45.2 kPa correlating with favorable outcomes (15), while Wang et al. identified an optimal therapeutic window of 132–162 kPa (converted from 1.3–1.6 atm) for balancing efficacy and morbidity (16). Notwithstanding these insights, current clinical practice remains constrained by two critical limitations: 1) commercial catheter systems lack integrated manometry capabilities, and 2) surgeons rely on subjective pressure estimation without quantitative benchmarks. This evidence gap necessitates the implementation of precision intraoperative manometry to establish robust pressure-efficacy-safety correlations and define phase-specific therapeutic thresholds—imperative advancements for standardizing PBC protocols and optimizing patient outcomes.

This study employed a custom-developed manometry system engineered by our research team to monitor intraoperative balloon pressure dynamics in real-time. Post-procedural analysis characterized pressure trajectory profiles, enabling phase-based classification according to morphological transitions. Subsequent correlation analyses examined relationships between phase-specific pressure parameters and postoperative complications, thereby generating clinical and theoretical evidence to advance precision medicine in trigeminal neuralgia management.

## 2 Material and methods

### 2.1 Study population

**Figure 1:**
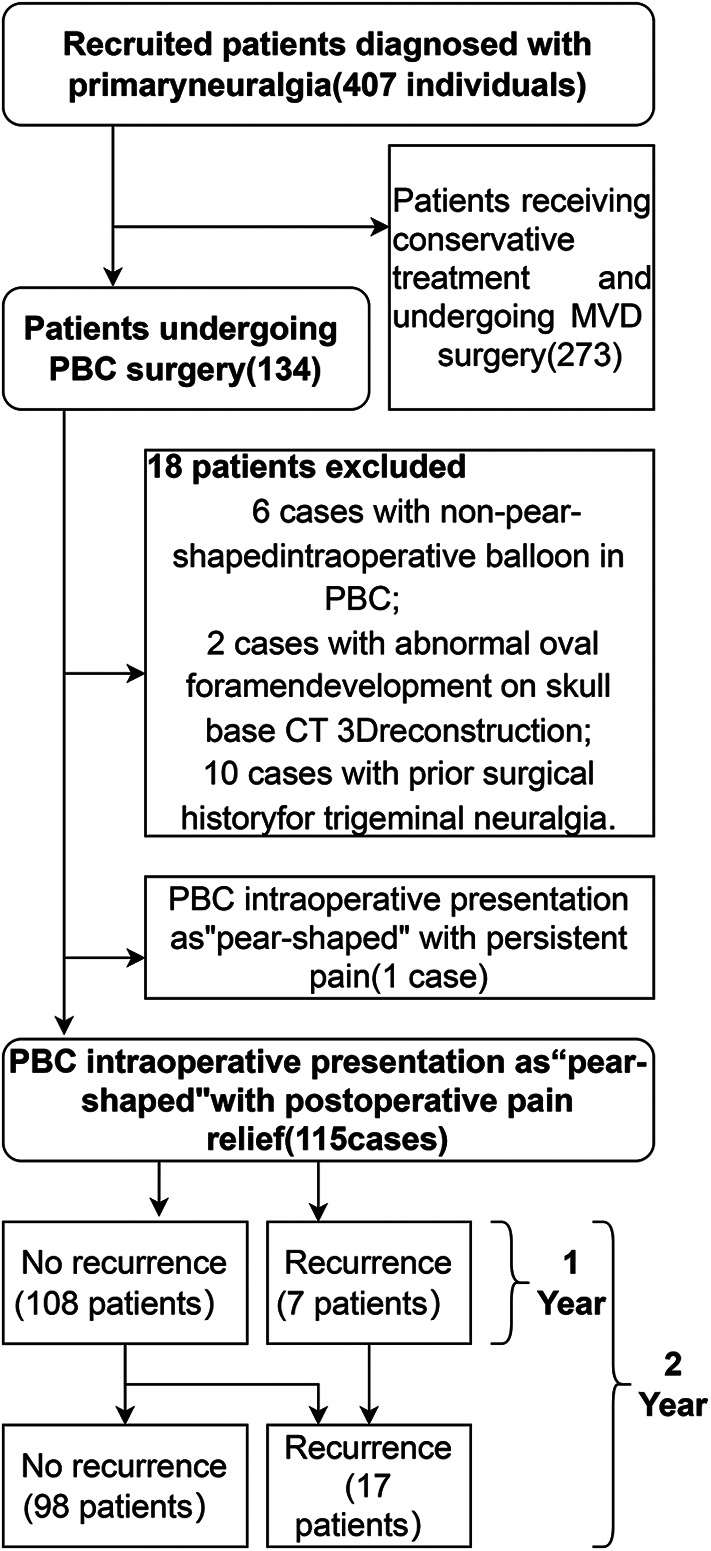
Flowchart of inclusion and exclusion criteria.

A prospective cohort of 115 patients with primary trigeminal neuralgia (TN) underwent percutaneous balloon compression (PBC) at the Department of Neurosurgery, Gansu Provincial People's Hospital between January 2017 and August 2024 (Figure 1).

#### 2.1.1 Inclusion criteria

Diagnosis of primary TN according to the Chinese Expert Consensus on Trigeminal Neuralgia, aligning with ICHD-3 diagnostic criteria (code 13.1.1.1); Failure or intolerance to ≥6 months of pharmacological therapy (carbamazepine/oxcarbazepine first-line) with contraindications to or refusal of open cranial surgery; Intraoperative confirmation of pear-shaped balloon morphology; Provision of written informed consent by patients and legal guardians.

#### 2.1.2 Exclusion criteria

Skull base CT 3D-reconstruction demonstrating foramen ovale malformation or positional anomalies; Secondary TN ruled out by contrast-enhanced cranial MRI; Comorbid craniofacial neurological disorders or severe cardiopulmonary dysfunction (NYHA Class III/IV or GOLD Stage 3/4); Preoperative ECG or echocardiography revealing significant arrhythmias or severe valvular defects.

The study protocol complied with the Declaration of Helsinki (2013 revision) and received ethical approval from the Institutional Review Board of Gansu Provincial People's Hospital (Approval No: 2022-072). Written informed consent was obtained from all participants.

### 2.2 Operative technique

**Figure 2:**
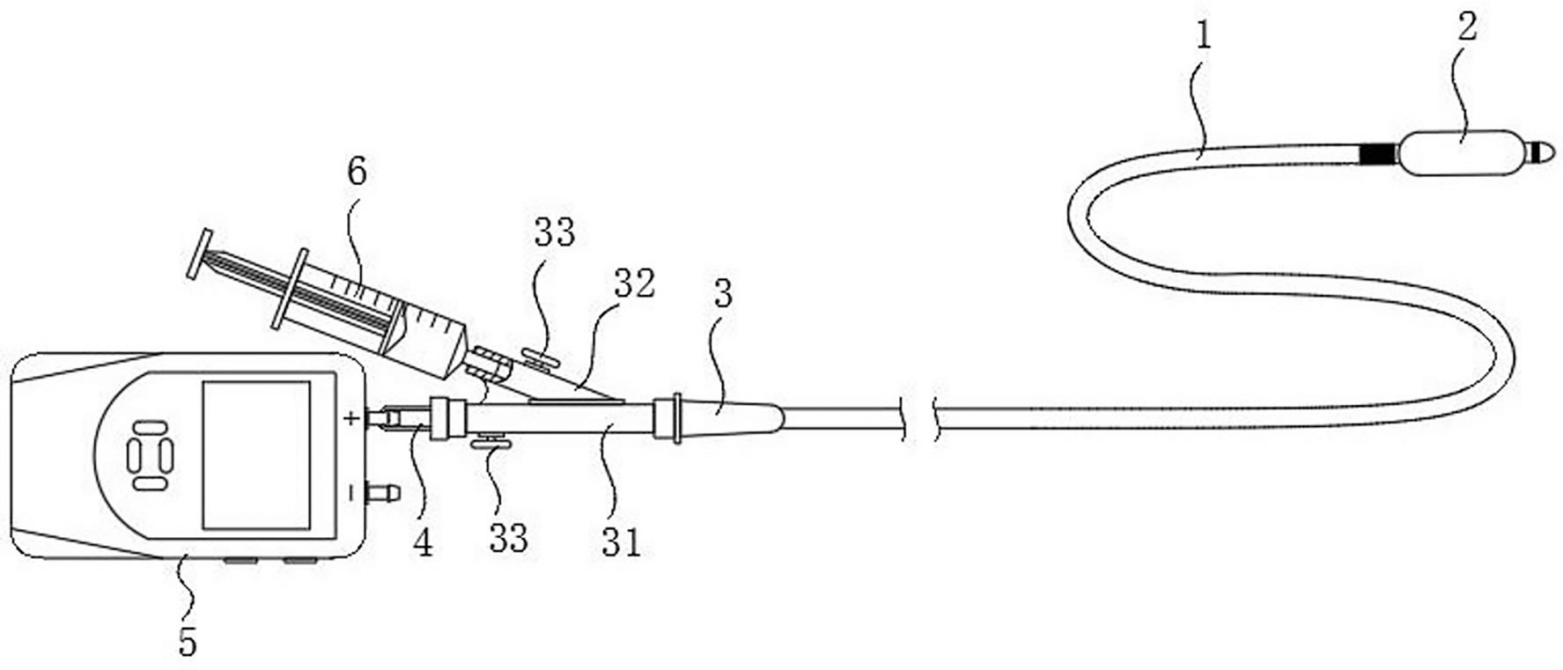
Surgical pressure testing instrument diagram. 1. Balloon catheter 2. Balloon 3. Balloon connector 31, 32. The medical three - way tubes 4. Infusion set connector 5. Manometer 6. Syringe.

Before surgery, remove the inner core of the balloon catheter (a single - use neurosurgical balloon catheter kit, model QKS - 0050005, from Shenzhen Qingyuan Medical Instruments Co., Ltd.). Connect its end to the front port of a three - way tube. Link the three - way tube's side port to a manometer (an AZ8230 digital pressure gauge from Taiwan's Heng Hsin Company), and connect the gauge to computer recording software (AZInstrumentCorp, handheld instrument data - logging software, version 3.10). Attach a 2.5 - mL syringe containing 2.0 mL iohexol to the rear port of the three - way tube. Inject about 1 mL iohexol into the balloon catheter to check the balloon's integrity. Repeatedly inflate and aspirate several times to remove air from the balloon, and set it aside for later use (Figure 2).

**Figure 3:**
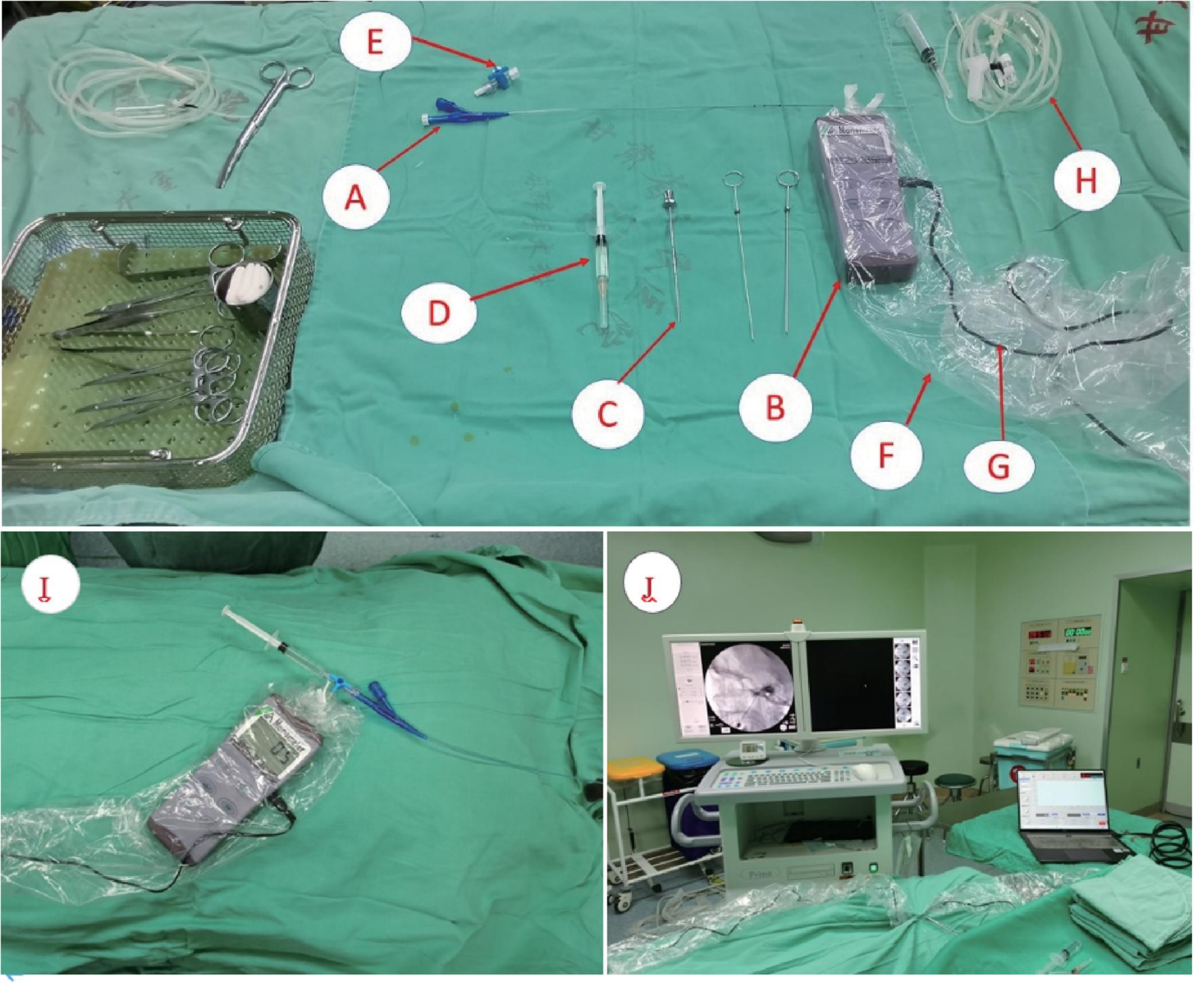
The components required for the intraoperative pressure measurement setup. **(A)** Medical three-way stopcock. **(B)** Pressure transducer. **(C)** Puncture needle. **(D)** Syringe. **(E)** Balloon catheter. **(F)** disposable laparoscopic set. **(I)** The connection of the pressure transducer, three-way stopcock, balloon catheter, and syringe. **(J)** Equipment and computer manometric software connected for intraoperative fluproscopy.

All procedures were performed under monitored anaesthesia care with endotracheal intubation. Patients were positioned supine with continuous electrocardiographic and pulse oximetry monitoring. A modified Hartel anterior approach was utilized for foramen ovale cannulation. The entry point was located 2.5–3.0 cm lateral to the oral commissure on the affected side, directed medially toward the mid-pupillary line and posteriorly toward a point 3 cm anterior to the external auditory meatus. After skin puncture with a 14-gauge needle, the cannula was advanced under fluoroscopic guidance. Upon confirming correct positioning within the foramen ovale, the cannula was advanced ∼1 cm intracranially. The stylet was then withdrawn, and the balloon catheter tip inserted through the cannula sheath into Meckel's cave along the mandibular nerve (V3). Under real-time fluoroscopy: 0.2 mL iohexol was injected to initiate balloon inflation. Additional 0.2–0.5 mL iohexol was administered until optimal pear-shaped morphology was achieved (17). Pressure recording commenced (1 Hz sampling) with the syringe barrel fixed. Upon detecting a > 30% pressure drop, supplemental 0.1–0.2 mL iohexol was injected to restore baseline pressure ±10%. The syringe barrel was re-fixed until compression completion. Balloon compression was maintained for 150 s in all cases. The cannula was withdrawn with 5-minute manual compression at the puncture site. Perioperative management: Atropine (0.5 mg IV) was prepared for trigeminocardiac reflex prophylaxis. Postoperative antiviral prophylaxis (e.g., acyclovir 400 mg TDS) prevented perioral herpes. Ocular lubricant ointment was applied to mitigate keratopathy (Figure 3).

### 2.3 Balloon pressure phases

Given uniform 150-second compression duration across all cases, pressure dynamics were classified into three consecutive phases:

Pear-Shaping Phase Initiated when fluoroscopy confirmed optimal pear-shaped morphology. Terminated upon detection of a rapid pressure decline (defined as >15% drop within 5 s). During this phase: Mechanical deformation of dural layers within Meckel's cave occurs, facilitating ganglion compression.

Pressure range was recorded at 1-second intervals. 2. Pressure-Maintenance Phase Commencing after pear-shaping phase completion: Supplementary iohexol (0.1–0.2 mL) was administered to elevate pressure to 20%–30% above the pear-shaping baseline. Continuous micro- injections maintained pressure within ±10% of target value. Pressure decay slope was quantified.

Full-Compression Phase: Encompassing the entire 150-second procedure from initial inflation to decompression. Integrated pressure metrics included: Time-weighted mean pressure Area under pressure-time curve (AUC).

### 2.4 Balloon pressure monitoring

**Figure 4:**
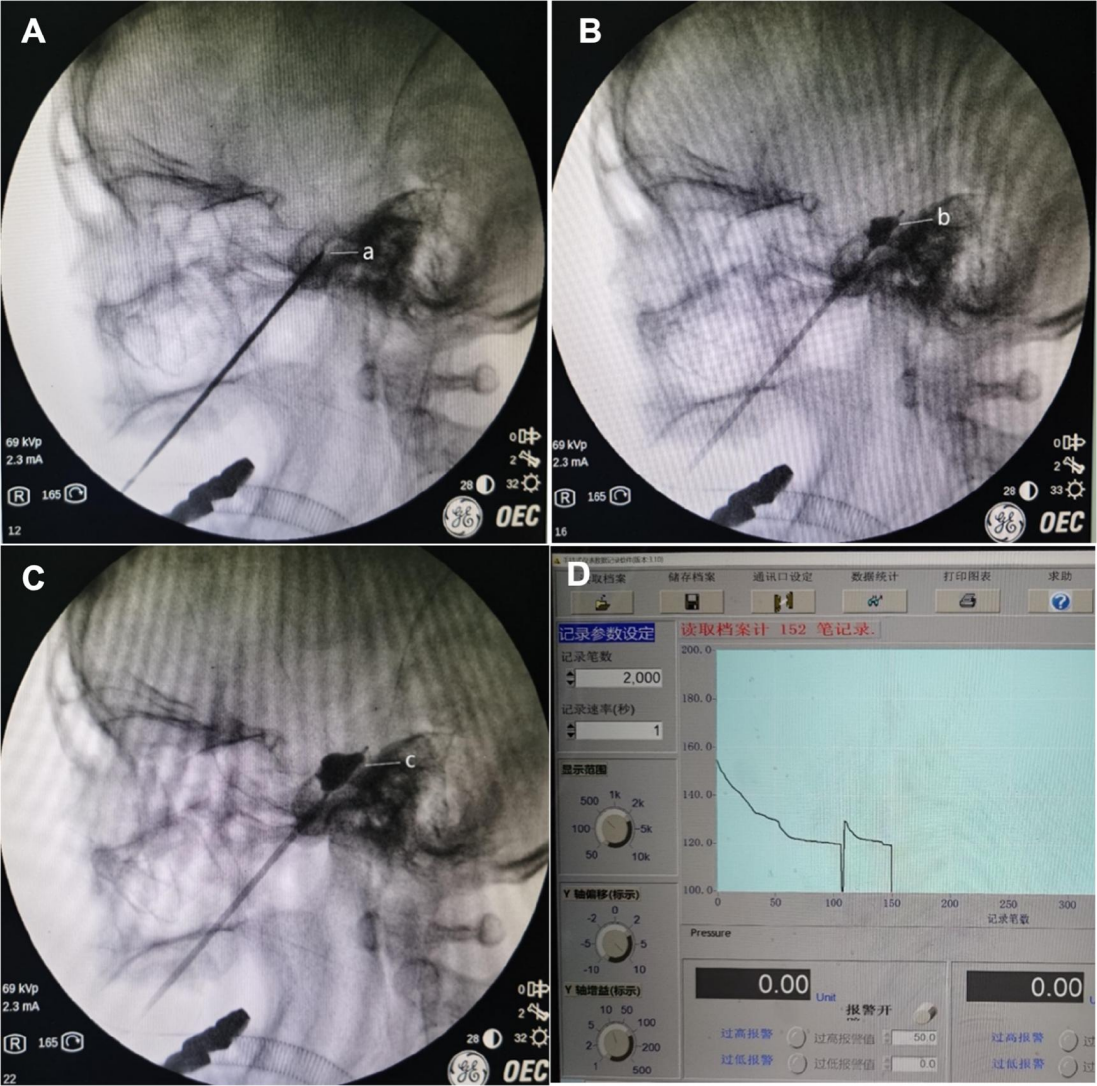
Balloon shape changes during PBC puncture and corresponding pressure changes in the manometer. **(A)** Puncture needle reaches the foramen ovale; **(B)** during the procedure, the balloon takes on an "under-inflated pear shape"; **(C)** during the procedure, the balloon forms the ideal "pear shape"; **(D)** postoperative trend of reorganized balloon pressure.

The full-compression phase (150 s in all cases, consistent with institutional protocol) spanned from initial pear-shaped formation to decompression. Pressure dynamics were stratified into two distinct intervals: Pear-Shaping Phase Initiation: Fluoroscopic confirmation of ideal pear-shaped morphology Termination: Objective detection of rapid pressure decay (operationalized as >15% decrease within 5 s) Biomechanical Events: Transient dural deformation within Meckel's cave enabling effective ganglionic compression Data Capture: Continuous pressure recording at 1 Hz sampling frequency Pressure-Maintenance Phase Commencement: Immediate post pear-shaping phase Pressure Modulation: Bolus injection of 0.1–0.2 mL iohexol to achieve 120%–130% baseline pressure recovery Microadjustments maintaining pressure within ±10% target threshold Mechanical Objective: Sustain axonal compression via controlled meningeal displacement Quantitative Analysis: Linear regression of pressure decay gradient (kPa/s) (Figure 4).

### 2.5 Statistical determination of pressure reference intervals

In this study, the pressure ranges for the balloon's pyriform phase, maintenance phase, and the entire procedure were calculated using the non-parametric method specified in the CLSI C28-A2 document. Initially, we excluded balloon pressure data from patients who experienced unrelieved pain 24 h post-surgery or had pain recurrence at 12 months. The remaining data were sorted in ascending order, denoted as X₁, X₂, X₃, .., X_n−1_, X_n_. Here, *P* represents the rank of each data point (*P* = 1∼n), with a sample size of *n* (*n* > 120). To determine the reference ranges, we calculated the ranks for the lower limit, *P* (0.5) = 0.005 × (*n* + 1), and the upper limit, *P* (99.5) = 0.995 × (*n* + 1). The corresponding balloon pressures for these ranks, *P*_0.5_ and *P*_99.5_, established the reference range as P_0.5_ to *P*_99.5_. Additionally, we used the Kolmogorov–Smirnov test to assess the distribution of time data for the pyriform and maintenance phases, checking for uniformity and calculating the mean time and fluctuation range (mean ± standard deviation) to clearly define the time frames of these two phases. In the results section, the measured data were presented directly.

### 2.6 Relevant assessment criteria

#### 2.6.1 Assessment of pain

Postoperative pain relief was evaluated at 24 h using the Barrow Neurological Institute (BNI) Pain Intensity Score (18): Grade I: Pain-free without medication, Grade II: Occasional non-disabling pain, Grade III: Pain adequately controlled with medication, Grade IV: Significant pain refractory to medication, Grade V: Severe intractable pain, Treatment response was defined as: Effective: BNI Grades I–II Ineffective: BNI Grades III–V (Table 1). Pain recurrence criteria required: BNI Pain Intensity ≥ Grade II at the 12-month or 24 months follow-up visit. Subsequent scheduled assessments were performed at 12 and 24 months. All evaluations were conducted by consultant-level neurologists (minimum ten years’ experience); any discrepancies were resolved by a third blinded assessor.

**Table 1:** Barrow Neurological Institute (BNI) pain intensity grade.

Grade	Description
I	No pain
II	Occasional pain
III	Pain controlled with medication
IV	Pain not controlled with medication
V	Severe pain unrelieved

#### 2.6.2 Assessment of hypoesthesia

Facial hypoaesthesia was evaluated at 24 h and 12 months postoperatively using the BNI Facial Hypoaesthesia Scale: Grade I: No sensory deficit, Grade II: Mild hypoaesthesia without functional impairment, Grade III: Moderate hypoaesthesia affecting daily activities, Grade IV: Severe disabling hypoaesthesia, Clinically significant hypoaesthesia was defined as Grades II–IV. Discrepancies underwent adjudication by a third blinded consultant. Follow-up combined outpatient reviews with structured telephone interviews by neurosurgeons trained in standardized assessment protocols (Table 2).

**Table 2:** Assessment of facial numbness using BNI facial numbness intensity grading.

BNI grade	Description	Facial numbness occurrence
I	No pain	No
II	Occasional pain	Yes
III	Pain controlled with medication	Yes
IV	Pain not controlled with medication	Yes

### 2.7 Statistical analysis

All analyses were performed using IBM SPSS Statistics 25.0 and R 4.0.3 under a predefined analytical protocol. Descriptive statistics characterized patient demographics, surgical history, and complication profiles, with intergroup comparisons (recurrence vs. non-recurrence) contingent upon distributional properties assessed by Kolmogorov–Smirnov tests. Normally distributed continuous variables underwent independent samples t-tests, while non-normal continuous variables and categorical data were analyzed via Mann–Whitney U tests or chi-square/Fisher's exact tests respectively. Spearman's rank correlation (*ρ*) quantified associations between intraoperative balloon pressure metrics and both postoperative hypoaesthesia severity and pain recurrence risk. Cox proportional hazards regression with Schoenfeld residual validation identified independent predictors of complications and recurrence, with hazard ratios (HRs) reported at 95% confidence intervals. Statistical significance was defined as two-tailed *P* < 0.05, with all methodologies conforming to TRIPOD and STROBE reporting guidelines.

## 3 Results

### 3.1 Comparison of patient baseline characteristics between normal and recurrence groups

Comparative analysis at 12 months demonstrated significantly higher intraoperative pressures in the non-recurrence cohort (*n* = 108) versus recurrence cohort (*n* = 7): full-compression phase (131.0 kPa [IQR 129–134] vs. 125.0 kPa [122–128]; *P* < 0.001), pear-shaping phase (134.0 kPa [131–138] vs. 126.0 kPa [126–129]; *P* = 0.001), and maintenance phase (125.0 kPa [123–126] vs. 123.0 kPa [116–123]; *P* = 0.039). Symptom duration was prolonged in recurrence cases (25.0 months [24.5–31.5] vs. 20.0 [13.8–25.0]; *P* = 0.017). No significant intergroup differences (*P* > 0.05) were observed in BNI hypoaesthesia scores (24 h/12mo), sex distribution, age stratification, lesion topography, or trigeminal branch involvement. These findings suggest phase-specific pressure metrics and symptom duration may serve as predictors of recurrence, whereas sensory outcomes and demographic/anatomic factors demonstrate limited prognostic utility (Table 3).

**Table 3:** Comparison of patient baseline characteristics between normal and recurrence groups (12 months).

Variable	Non-recurrence group	Recurrence group	*p*.overall
	*N* = 108	*N* = 7	
The whole period pressure	131 [129;134]	125 [122;128]	<0.001
Pears shaped period pressure	134 [131;138]	126 [126;129]	0.001
Maintenance period pressure	125 [123;126]	123 [116;123]	0.039
Postoperative numbness score			0.553
(12 month)			
1	44 (40.7%)	4 (57.1%)	
2	61 (56.5%)	3 (42.9%)	
3	3 (2.78%)	0 (0.00%)	
Gender			1
Male	44 (40.7%)	3 (42.9%)	
Female	64 (59.3%)	4 (57.1%)	
Age:			0.571
≤65 years	15 (13.9%)	1 (14.3%)	
65–70 years	42 (38.9%)	4 (57.1%)	
≥70 years	51 (47.2%)	2 (28.6%)	
Lesion location			1
Right	47 (43.5%)	3 (42.9%)	
Left	61 (56.5%)	4 (57.1%)	
Onset duration	20.0 [13.8;25.0]	25.0 [24.5;31.5]	0.017
Distribution of trigeminal neuralgia			0.92
V1	2 (1.85%)	0 (0.00%)	
V2	19 (17.6%)	1 (14.3%)	
V3	30 (27.8%)	2 (28.6%)	
V1 + V2	6 (5.56%)	0 (0.00%)	
V1 + V3	1 (0.93%)	0 (0.00%)	
V2 + V3	37 (34.3%)	4 (57.1%)	
V1 + V2 + V3	13 (12.0%)	0 (0.00%)	

Comparative assessment at 24 months revealed significantly elevated intraoperative pressures in the non-recurrence cohort (*n* = 98) versus recurrence group (*n* = 17) for both full-compression (*P* < 0.001) and pear-shaping phases (*P* < 0.001), whereas maintenance phase pressures demonstrated no intergroup difference (*P* = 0.289). Symptom duration remained prolonged in recurrence cases (*P* = 0.014). No significant associations were observed between recurrence status and: BNI hypoaesthesia scores at 24 h (*P* = 0.396) or 12mo (*P* = 0.308) Sex distribution (*P* > 0.99) Age stratification (*P* > 0.99) Lesion topography (*P* = 1.000) Trigeminal branch involvement (*P* = 1.000). These findings reinforce phase-specific pressure thresholds (full-compression/pear-shaping) and symptom duration as potential predictors of long-term recurrence, contrasting with the limited prognostic utility of sensory outcomes, demographic variables, and anatomical factors (Table 4).

**Table 4:** Comparison of patient baseline characteristics between normal and recurrence groups (24 months).

Variable	Non-recurrence group	Recurrence group	*p*.overall
	*N* = 98	*N* = 17	
The whole period pressure	131 [129;134]	128 [125;131]	<0.001
Pears shaped period pressure	134 [131;138]	128 [126;131]	<0.001
Maintenance period pressure	124 [123;126]	124 [121;126]	0.289
X24 month postoperative numbness score			0.308
1	38 (38.8%)	10 (58.8%)	
2	57 (58.2%)	7 (41.2%)	
3	3 (3.06%)	0 (0.00%)	
Gender			1
Male	40 (40.8%)	7 (41.2%)	
Female	58 (59.2%)	10 (58.8%)	
Age			1
≤65years	14 (14.3%)	2 (11.8%)	
60–70years	39 (39.8%)	7 (41.2%)	
≥70 years	45 (45.9%)	8 (47.1%)	
Lesion location			1
Right	43 (43.9%)	7 (41.2%)	
Left	55 (56.1%)	10 (58.8%)	
Onset duration	19.0 [13.0;24.0]	25.0 [23.0;28.0]	0.014
Distribution of trigeminal neuralgia			0.99
V1	2 (2.04%)	0 (0.00%)	
V2	17 (17.3%)	3 (17.6%)	
V3	27 (27.6%)	5 (29.4%)	
V1 + V2	5 (5.10%)	1 (5.88%)	
V1 + V3	1 (1.02%)	0 (0.00%)	
V2 + V3	34 (34.7%)	7 (41.2%)	
V1 + V2 = V3	12 (12.2%)	1 (5.88%)	

### 3.2 Balloon pressure dynamics in case ID 001

**Figure 5:**
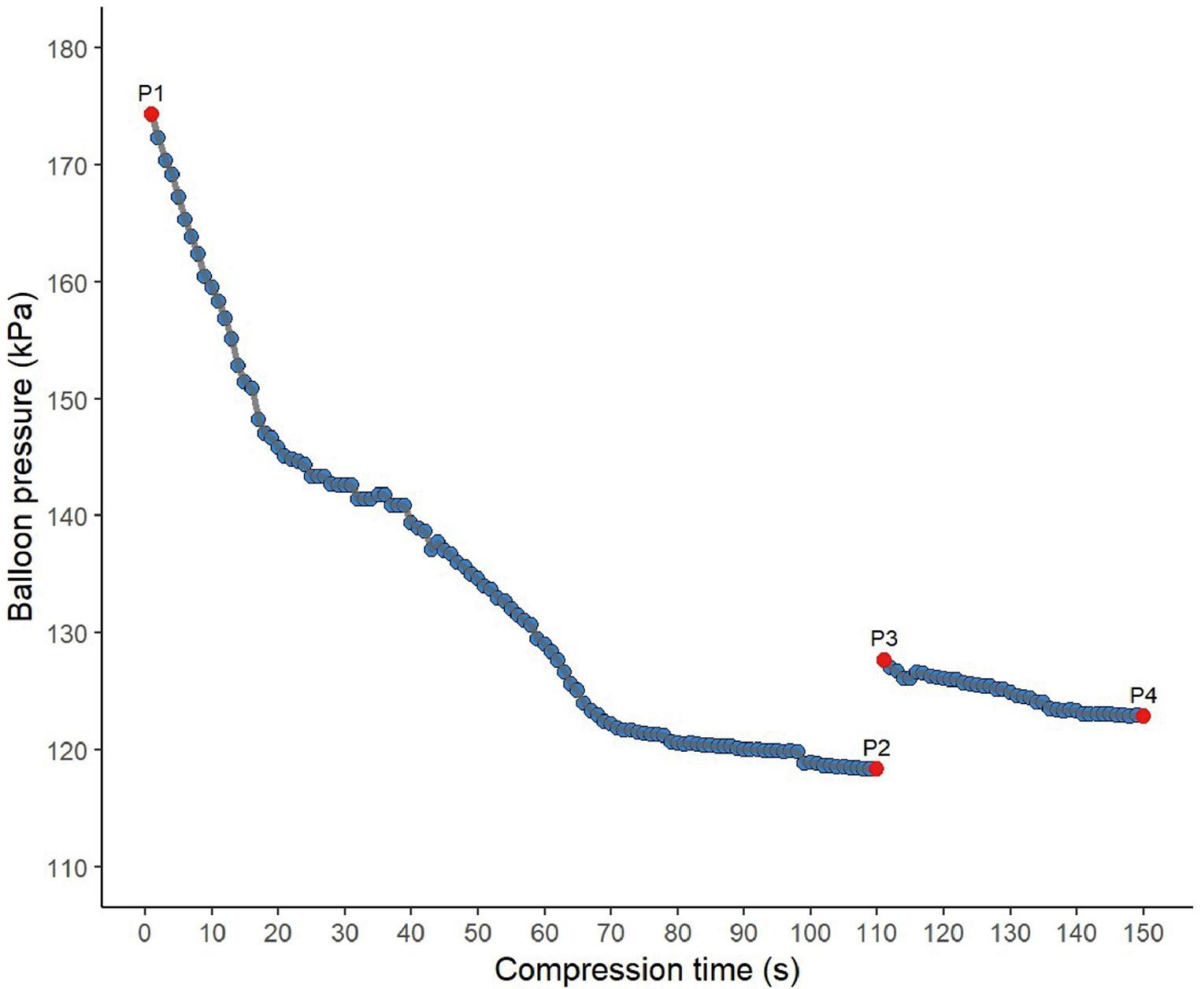
Balloon pressure dynamics in case ID 001.

Intraoperative monitoring revealed a distinct biphasic profile: the pear-shaping phase (0–109.0 s) transitioned to the maintenance phase (109.0–150.0 s) following abrupt pressure decay (>15%/5 s). Therapeutic pressure was restored via contrast boluses (0.1–0.2 mL), eliciting rapid recovery (*Δ* > 30 kPa within 1 s) followed by gradual decline (slope: −0.8 kPa/s). As illustrated in Figure 5A, key inflection points included: initial inflation peak (P1: 175 kPa), early decay plateau (P2: 142–155 kPa), phase transition threshold (P3: 125 kPa), and maintenance band (P4: 110–130 kPa) (Figure 5).

### 3.3 Pressure reference intervals

Phase-specific reference intervals were established per CLSI EP28-A3c guidelines: Pear-shaping phase (*n* = 10,641 measurements):125.7–157.6 kPa Maintenance phase (*n* = 4,059 measurements):116.0–134.4 kPa Full-compression phase (*n* = 14,700 measurements):118.9–146.3 kPa These evidence-based thresholds provide quantitative benchmarks for intraoperative balloon pressure regulation during percutaneous trigeminal ganglion compression (Table 5).

**Table 5:** Pressure reference intervals.

Variable	Pear-shaped stage	Maintenance pase	Whole course phase
Observed values	10,641	4,059	14,700
Reference intervals	157.6∼125.7	134.4∼116.0	146.3∼118.9

### 3.4 Analysis of the correlation between balloon pressure in different postoperative stages and facial complications

**Figure 6:**
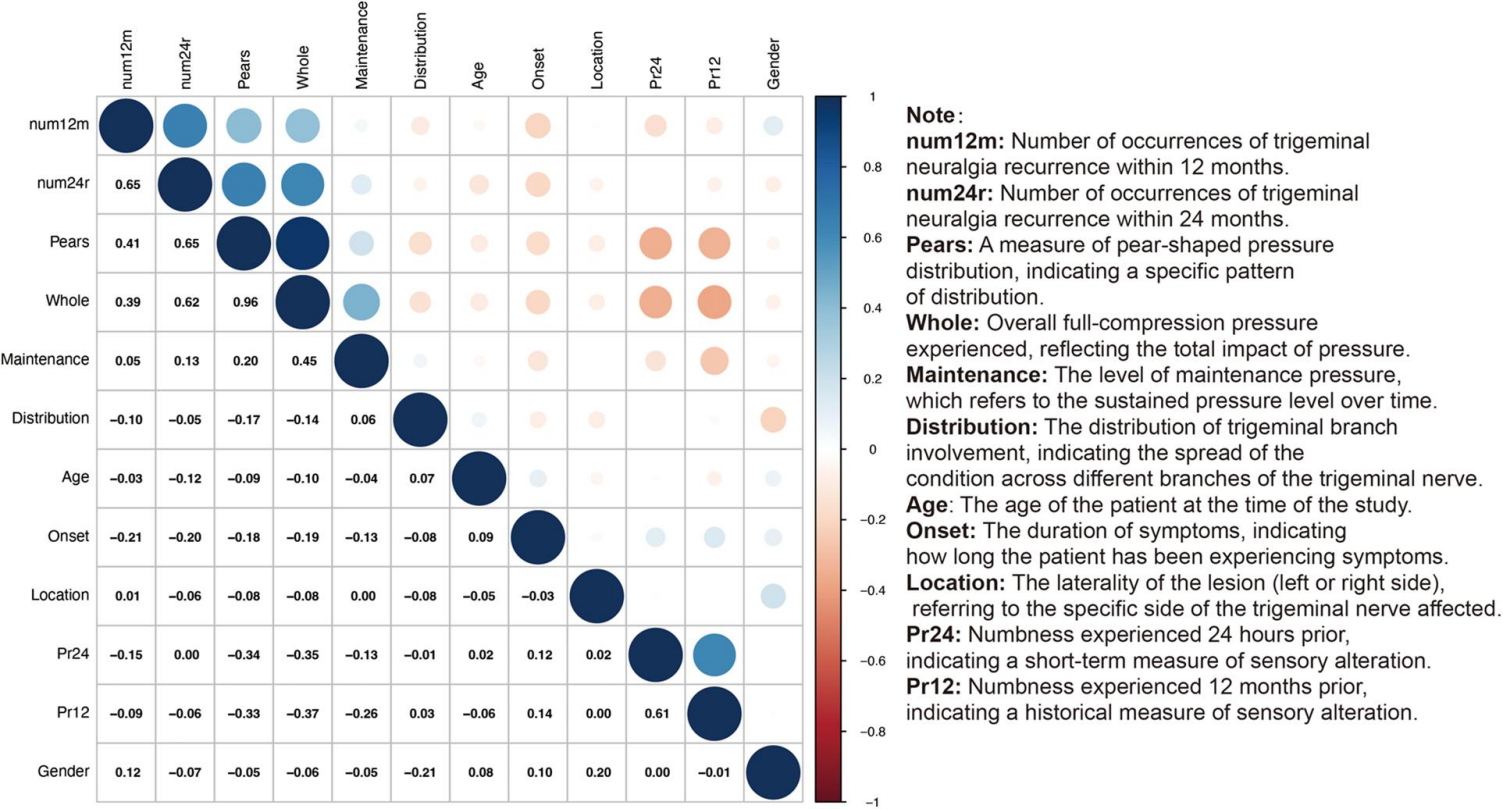
Analysis of the correlation between balloon pressure in different postoperative stages and facial complications.

The analysis revealed a significant positive correlation between the number of trigeminal neuralgia recurrences at 12 months (num12 m) and at 24 months (num24r) (r = 0.65), indicating a persistent pattern of recurrence over time. The overall recurrence rate (Whole) also showed a high positive correlation with the recurrence rates at both 12 months (r = 0.39) and 24 months (r = 0.62), further confirming the consistency of the condition's impact. Furthermore, we assessed the relationship between paresthesia at different time points and the recurrence rate. We found that paresthesia experienced 24 h prior (Pr24) and paresthesia experienced 12 months prior (Pr12) both had some correlation with the recurrence rate, which may suggest that paresthesia is a predictive factor for the recurrence of trigeminal neuralgia. Notably, the slight negative correlation between paresthesia experienced 12 months prior (Pr12) and the laterality of the lesion (Location) (r = −0.08) might reflect the impact of lesion location on the experience of paresthesia. During the maintenance phase, we observed a moderate positive correlation with the recurrence rates at 12 months (r = 0.05) and 24 months (r = 0.13), which could imply a potential role for maintenance therapy in controlling the recurrence of the condition, although the correlation is not strong. No significant correlation was found between the distribution of recurrence rates and other variables, indicating a relatively uniform distribution of recurrences across different subgroups. In summary, our findings reveal patterns of trigeminal neuralgia recurrence and its relationship with paresthesia, providing a new perspective for understanding the long-term management and treatment of this complex condition. These insights could potentially influence the development of clinical treatment strategies, especially in considering how maintenance therapy might be used to reduce recurrences and improve patients' quality of life (Figure 6).

### 3.5 The non-recurrence curve of patients (grouped by sex and age)

**Figure 7:**
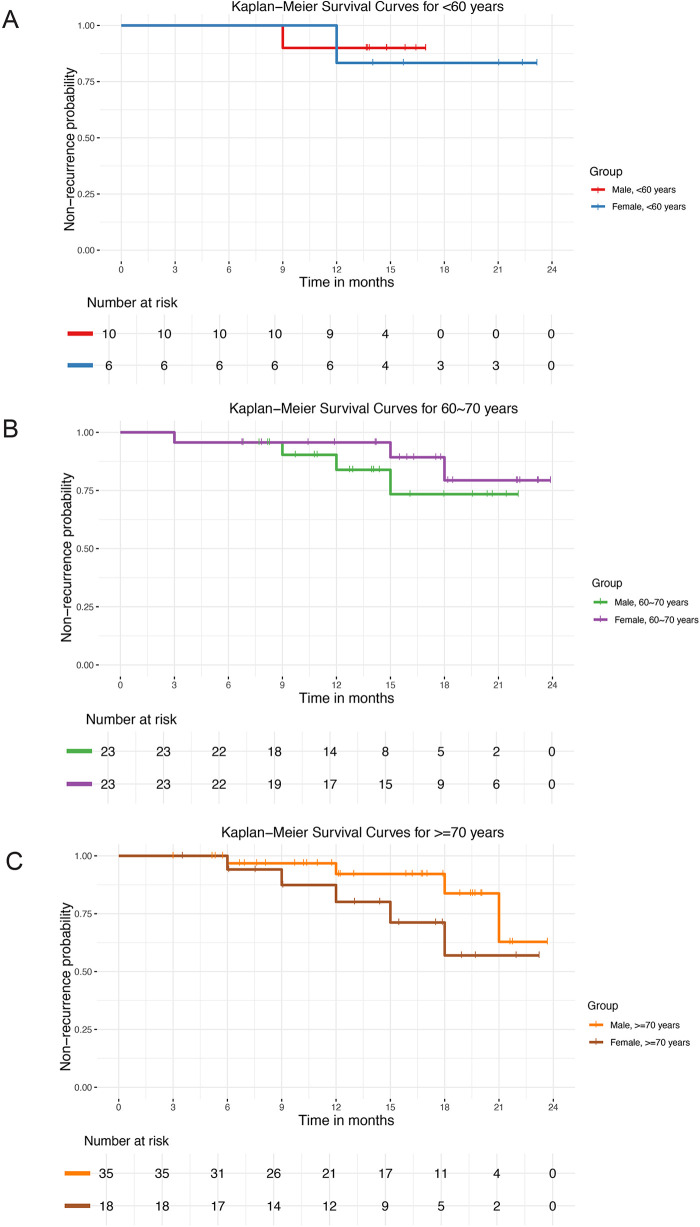
Kaplan-Meier survival curves showing non-recurrence probability by sex across different age groups. **(A)** Patients <60 years. **(B)** Patients 60–70 years. **(C)** Patients ≥70 years.

We constructed the Kaplan–Meier curves of postoperative pain recurrence using time to postoperative pain recurrence as the horizontal axis and postoperative non-recurrence rate as the vertical axis. The curves were grouped by gender and age group. The risk table illustrated the precise number of individuals at risk of postoperative pain recurrence with increasing postoperative time. The Kaplan–Meier curves indicated that the recurrence rate was higher in men than in women in the younger below 60 years (Figure 7A) and older above 70 years (Figure 7C) age groups. Conversely, the recurrence rate was higher in women than in men in the intermediate age group 60–70 years (Figure 7B). However, this difference was not statistically significant.

## 4 Discussion

Since its first report in 1983, PBC has been refined and is now an effective minimally invasive treatment for TN (19). By mechanically damaging the trigeminal nerve's sensory fibers, it alleviates pain. Surgical factors like balloon shape, compression time, and pressure significantly affect outcomes (9, 20). Our study highlights that compression time and pressure are especially critical. Intraoperative lateral X - ray fluoroscopy showing a balloon in a “pear - shaped” form is the gold standard for confirming accurate placement in Meckel's cavity (13, 16, 21). All patients in this study met this standard, with 99.1% achieving pain relief, indicating a highly effective procedure. A 150 - second compression time ensures efficacy while preventing significant facial numbness - related quality - of - life impacts. We assessed facial numbness at various postoperative time points. At 24 h post - surgery, among 115 pain - relieved patients, there were 19, 63, 33, and 0 patients with Grade Ⅳ, Ⅲ, Ⅱ, and Ⅰ facial numbness, respectively. At 12 months, these numbers were 0, 3, 64, and 48. The proportion of Grade Ⅳ facial numbness at 24 h was 16.52%, but at 12 months, Grade Ⅰ and Ⅱ cases accounted for 97.3%, with only 3 patients having Grade Ⅲ or higher facial numbness. This shows that facial numbness decreases over time with minimal impact on patients’ lives, aligning with prior studies that PBC is effective and has few complications when compression time is within 1–3 min. After recognizing the importance of balloon shape and compression time in PBC surgery, we delved deeper into how balloon pressure affects treatment and post - operative complications.

Our study found that balloon pressure during the pear - shaped, holding, and entire periods is closely linked to the incidence and severity of facial numbness - the higher the pressure, the higher the numbness grade. Looking at 19 patients with Grade Ⅲ or higher facial numbness at 24 h post - surgery, we found their initial pear - shaped phase pressure all exceeded 170 kPa. This shows controlling pressure during this phase is key as excessive initial pressure can lead to severe facial numbness, so initial pear - shaped phase pressure should be limited. Also, balloon pressure is negatively related to pain recurrence risk - the lower the pressure, the higher the risk. In our study, the 3 recurrence cases had a history of multiple prior surgeries at other hospitals and their initial balloon pressure was all below 141 kPa. We guess severe Meckel's cavity adhesions might reduce the lesion effect at low pressure, so properly increasing pressure during the holding phase may enhance therapeutic effect. Moreover, the lesion side and patient age showed no significant link to post - operative outcomes. This indicates that, when the balloon takes a “pear shape”, pressure and time are key factors in determining the extent of trigeminal ganglion damage. Other studies have similar findings. Zanusso et al. (22) in a study of 22 patients, found under the same compression time (3.5 min), the low - pressure (0.9–1.3 Bar) group had a 75% recurrence rate but no complications, while the high - pressure (1.9–2.4 Bar) group had no recurrences but more complications. Brown and Pilitsis' (15) research shows 1.5 - minute balloon compression at 1,140–1,215 mmHg pressure reduces complications like masseter weakness but doesn't affect pain or recurrence rates. Peng et al. (23) followed up with 45 patients for 3 months and found that intraoperative BP of 138.65–153.90 KPa can effectively relieve pain and reduce complications. So, balloon pressure is a key factor affecting PBC's efficacy and complications. Optimizing pressure control is of great importance for improving surgical outcomes and lowering recurrence and complication risks.

When exploring the factors affecting the post - operative outcome of percutaneous balloon compression (PBC), balloon pressure control is a vital part. Past studies indicate that intra - operative balloon pressure in PBC usually ranges from 60 to 170 kPa (24), 138.65 to 153.90 kPa (23), and 130 to 200 kPa (25). But these studies have limitations in guiding clinical practice. On the one hand, they fail to finely divide the surgical process based on the dynamic intra - operative intracystic pressure changes. On the other hand, the lack of continuous monitoring and real - time regulation of balloon pressure reduces the accuracy of pressure control, possibly affecting the stability and reproducibility of the surgical outcome. In this study, a real - time pressure monitoring system was innovatively introduced during PBC. By connecting a pressure transducer to a computer, balloon pressure changes were continuously recorded at a frequency of once per second. This provided crucial evidence for precise intra - operative balloon pressure regulation. Post - operatively, the research team rigorously analyzed the collected data, eliminating data from patients with unrelieved pain and recurrences that could cause interference. Then, following the robust assessment method recommended in the CLSI C28 - A2 document, reference ranges for balloon pressure in the pear - shaped phase, maintenance phase, and entire phase were determined as 126.5–156.8 kPa, 117.9–136.1 kPa, and 119.9–141.9 kPa, respectively. This staging and pressure control method based on real - time monitoring data helps surgeons precisely regulate balloon pressure during operations. It also effectively balances the risks of post - operative pain relief and complications, thus comprehensively optimizing patients' treatment outcomes and maximizing their benefits. Surgeons can more intuitively understand the dynamic changes in balloon pressure during surgery and adjust pressure parameters according to individual patients’ specific conditions. Moreover, robotic-assisted technology in peripheral nerve microsurgery provides an instructive paradigm for standardizing PBC. Research by Jiang et al. (26) demonstrated that in limb and cervicothoracic regions lacking natural anatomical spaces, the use of a robotic platform to create an artificial pneumocavity with integrated real-time pressure-visual feedback can reduce microsurgical error to sub-millimeter levels, limit incision length to less than 1 cm, and significantly lower perioperative complications such as nerve adhesion and scar-related pain. As early as 2016, the same team utilized the da Vinci system to establish an anterior cervical working cavity through bilateral 4-cm supraclavicular incisions and successfully performed contralateral C7-to-ipsilateral C7 nerve transfer in a cadaveric model (27).

The involvement of such advanced technology not only reduces surgical difficulty and operational risks but also minimizes tissue trauma and associated complications. The insight from Jiang et al. suggests that by enabling continuous digital monitoring of key intraoperative parameters—pressure, time, and spatial localization—procedures traditionally reliant on the surgeon's subjective “feel” can be transformed into repeatable, quality-controllable standardized workflows. Similarly, in PBC, real-time coupling of the “pear-shaped” configuration judgment with the balloon pressure-time curve, along with software-defined safety thresholds and alarms, could—much like the role played by the da Vinci platform in peripheral nerve surgery—elevate the principle of “sufficient yet not excessive” nerve ablation from an operator-dependent art to an algorithm-guided process. This shift would advance PBC from an experience-based practice toward precision medicine. Therefore, real-time balloon pressure monitoring is not only feasible but should be adopted as a routine component of PBC. Its potential contribution to surgical quality control parallels the successful paradigm established by robot-assisted minimally invasive techniques in the field of peripheral nerve surgery.

## 5 Limitation

Our single-center observational study has limitations that necessitate cautious interpretation. With a sample size of 115 patients and a maximum follow-up of 24 months, we may not fully capture long-term complications or recurrence trends. The observational design limits establishing a causal link between pressure parameters and outcomes, and we did not systematically collect all potential anatomical and pathological variables, which may affect the accuracy of our correlations. The accuracy of intraoperative pressure measurements is constrained by equipment resolution and may not fully capture pressure dynamics. Our inclusion criterion of a successful “pear shape” formation during the procedure means our findings apply mainly to this subgroup, with unknown differences in outcomes for those without this shape. Some outcome measures, like facial numbness, are subjective and may introduce bias. The single-center design may not reflect variations in surgical techniques across different centers, affecting the generalizability of our results. To overcome these limitations, we are initiating a prospective, multi-center study with at least 300 patients to collect detailed data and use advanced analytical methods to build a robust predictive model and better understand the relationship between pressure dynamics and clinical outcomes.

## 6 Conclusion

During PBC for trigeminal neuralgia, balloon pressure control is critical. We found that balloon pressure during the pear - shaped, holding, and entire periods is closely related to postoperative complications and recurrence. High pressure worsens facial numbness, and low pressure causes pain recurrence. The study gives reference ranges for balloon pressure in each stage: 126.5–156.8 kPa (pear - shaped), 117.9–136.1 kPa (holding), and 119.9–141.9 kPa (entire period). These offer a quantitative basis for intraoperative pressure control, helping optimise treatment and reduce complications. Also, the study highlights the value of real - time balloon pressure monitoring, which boosts the stability and reproducibility of the surgery, thus promoting the standardized and personalized development of PBC.

## Data Availability

The original contributions presented in the study are included in the article/Supplementary Material, further inquiries can be directed to the corresponding author.
